# Structural equation modelling for associated factors with dental caries among 3–5-year-old children: a cross-sectional study

**DOI:** 10.1186/s12903-019-0787-4

**Published:** 2019-06-06

**Authors:** Yuandong Qin, Rui Zhang, Bo Yuan, Ting Xu, Hong Chen, Yingming Yang, Tao Hu

**Affiliations:** State Key Laboratory of Oral Diseases & National Clinical Research Center for Oral Diseases & Dept. of Preventive Dentistry, West China Hospital of Stomatology, Sichuan University, No. 14, Sec.3, Ren Min South Road, Chengdu, Sichuan 610041 People’s Republic of China

**Keywords:** Dental caries, KAP, Structural equation model

## Abstract

**Background:**

The aim of the current study was to explore the factors influencing dental caries among 3–5-year-old children in Sichuan Province and the interrelationship between these factors using structural equation modelling (SEM).

**Methods:**

A cross-sectional study was conducted among 2746 3–5-year-old children in Sichuan Province. Examination of caries was conducted on all children and a questionnaire was answered by the children’s caregiver. SEM alternative models were constructed to interpret the intricate relationships between socio-economic status (SES), caregiver’s oral health knowledge, attitudes, children’s oral health behaviours and children’s dental caries.

**Result:**

The results showed that dental caries were significantly associated with dietary behaviours (β = 0.11, SE = 0.03, *P* = 0.001, BC 95% CI =0.05/0.18) and SES (β = − 0.17, SE = 0.03, *P*<0.001, BC 95% CI = -0.23/− 0.10) directly, While the indirect effect of SES on dmft is in an opposite direction (β = 0.08, SE = 0.02, BC 95% CI = 0.04/0.12).

**Conclusion:**

We found that unhealthy dietary behaviours increased the prevalence of dental caries. However, oral health knowledge and attitude failed to affect dietary behaviour in this model. This result warns that oral health education should strengthen feeding-related knowledge. Meanwhile, it also reminds that it is easier known than done. Future oral health education should focus on exploring a more effective way for the public to turn knowledge into action.

## Background

Dental caries is one of the most severe chronic diseases among children in both developed and developing countries [1]. In 2010, dental caries in deciduous teeth affect 621 million people worldwide, which is 9% of the global population [1]. Untreated dental caries may cause severe pain and mouth infection [2], which affects children’s school attendance and performance [3]. It is important to determine the risk factors associated with children’s dental caries, and to build effective prevention strategies.

It is recognized that dental caries is caused by a multiplicity of factors, including behaviours like tooth brushing and sugar intake [4, 5]. Conducting effective preventive behaviours relies on many factors. The knowledge-Attitude-Behaviour (KAB) model, which is developed as a health promotion model and frequently used to assess behaviour change, has been proposed as a way of explaining the role of knowledge [6]. It explains that a person’s knowledge directly affects attitudes, and indirectly affects behaviours through attitudes [6]. Oral health knowledge is considered to be an essential prerequisite for health-related behaviours [7]. Furthermore, improvement in knowledge has been known to influence not only self-reported oral health-related behaviours in a favourable way, but also improve clinical parameters of oral health such as oral hygiene, gingival health and dental caries [8, 9]. Moreover, oral health knowledge motivates positive attitudes to access information about oral health prevention and to perform oral health-related behaviours [10, 11]. Obtaining oral health knowledge and building a positive attitude to oral health are associated with socio-economic status (SES) [11]. Meanwhile, many epidemiological studies have demonstrated an association between SES and oral health in developed countries: lower SES groups have poorer oral health than higher ones [12, 13].

Many factors related to dental caries have been identified by multivariate regression in previous studies, but it is not clear whether these factors influence oral health directly or indirectly. Meanwhile, dental caries is a chronic disease influenced by various aspects simultaneously [2]. Therefore, it is important to study the multidimensional factors leading to dental caries simultaneously, and to determine effective intervention measures.

**Fig. 1:**
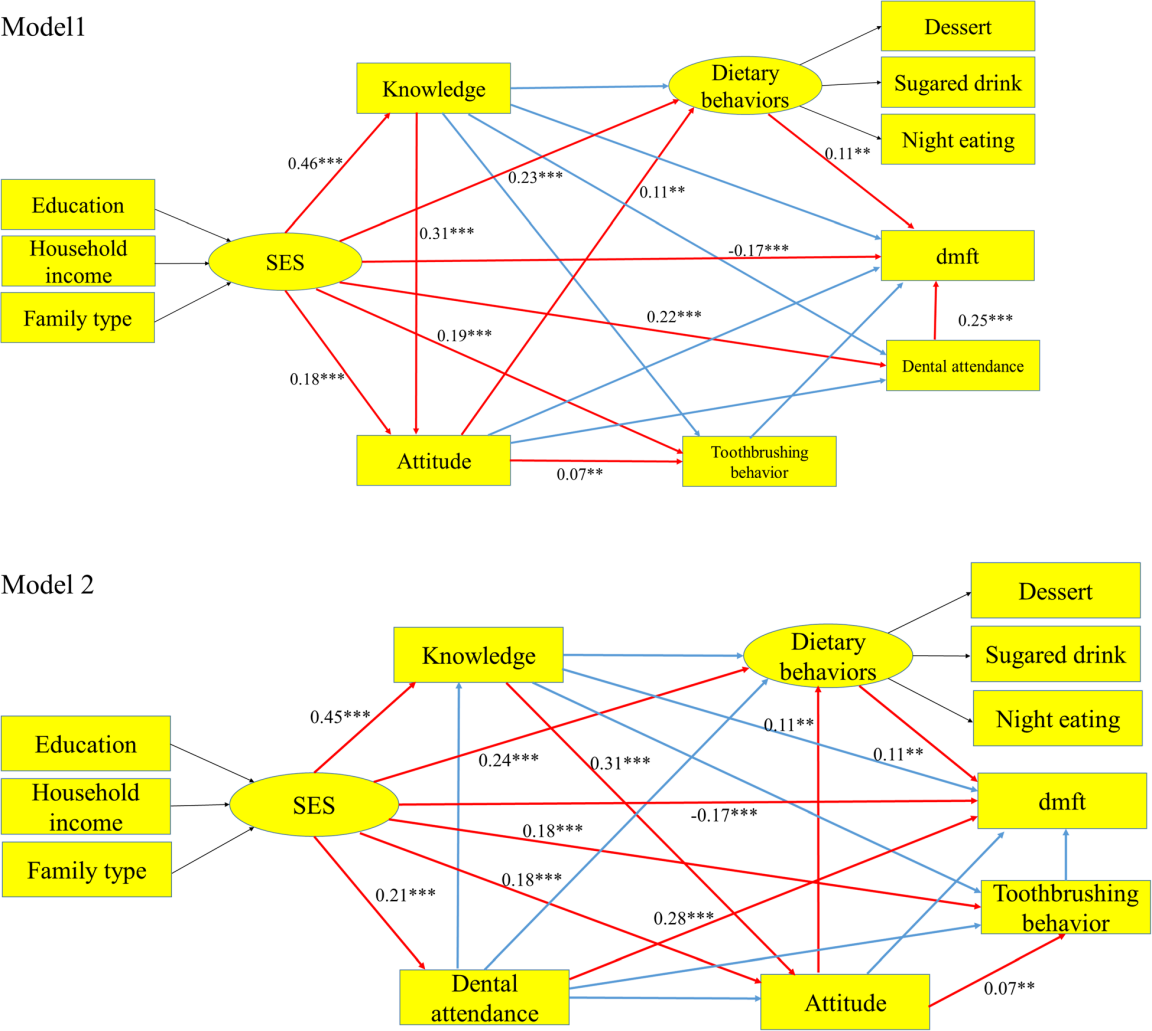
Standardized path coefficients of the hypothesized model 1 and model 2. Model 1: We assumed that dental caries (dmft) were directly influenced by SES, oral health knowledge, oral health attitudes, dietary behaviours, tooth brushing behaviours and dental attendance. Simultaneously, SES, oral health knowledge and oral health attitudes directly affected dietary behaviours and tooth brushing and dental attendance. Additionally, SES and oral health knowledge directly affected oral health attitudes, and SES directly influenced oral health knowledge. Model 2: based on the model 1, we assumed that oral health knowledge and attitude were directly affected by dental attendance and SES. Meanwhile, SES directly affected dental attendance. Red solid arrows mean significant effect while blue solid arrows indicate insignificant effect (the significant for path coefficients was set at 0.05, * *P* < 0.05, ** *P* < 0.01, ***P < 0.001)

Structural equation modelling (SEM) is an analytical technique for disentangling complex relationships and causal pathways when latent constructs are concerned [14]. To explore factors related to children’s dental caries and the direct or indirect relationship, we proposed to build alternative models of the complex relationship between SES, knowledge, attitudes, behaviour (including dietary behaviour, tooth brushing behaviour and dental attendance) and dental caries based on theoretical framework [11, 15] and literature using a SEM. Model 1:We assumed that dental caries (dmft) were directly influenced by SES [16], oral health knowledge, oral health attitudes [8, 17–19], dietary behaviours [20], tooth brushing behaviours [21] and dental attendance. Simultaneously, SES, oral health knowledge and oral health attitudes directly affected dietary behaviours and tooth brushing and dental attendance [22]. Additionally, SES and oral health knowledge directly affected oral health attitudes [11], and SES directly influenced oral health knowledge [11]. Therefore, we build a direct and indirect relationship network between the relative factors and dental caries (dmft). Model 2: based on the model 1, we assumed that oral health knowledge and attitude were directly affected by dental attendance and SES. Meanwhile, SES directly affected dental attendance, as shown in Fig. 1.

## Methods

### Sampling and sample sizes

Ethical approval was obtained from the Stomatological Ethics Committee of the Chinese.

Stomatological Association and the Ethics Committee of West China Hospital of Stomatology, Sichuan University (Approval No. 2014–003). A cross-sectional study was conducted among 3–5-year-old children in Sichuan Province. The sample size was calculated based on the oral disease prevalence derived from the Third National Oral Health Survey [23]. According to the formula below, the required sample size was 2472.

$$ \mathrm{N}=\mathrm{deff}\frac{\mu^2\left(1-p\right)}{\varepsilon^2p}/\left(1- non- responses\ rate\right) $$

N is the sample size, deff means design effect (2.5), *p* is the dental caries prevalence of 5-year-old children in the Third National Oral Health Survey because it only included 5-year-old children in the last nation oral health survey, which was 86.0%. μ = 1.96 and is the level of confidence, ε = 10% and is the margin of error, the non-responses rate was 20%. Hair [24] suggested that the minimum sample size for SEM was 500 for models including more than 7 constructs, and/or having fewer than three measured items. According to the estimation, we selected 2746 participants randomly to complete this study.

In this study, we obtained a representative sample by a multistage stratified cluster sampling method with selection probabilities proportional to size (PPS) [25]. Detail information regarding the sampling procedures had been published [26].

Before the study, all caregivers were provided with all details of the survey and signed informed consent were obtained.

### Data collect

Data was collected through questionnaire and oral examination at the participants’ kindergartens. Questionnaire study contained SES, caregiver’s oral health knowledge, attitudes and children’s oral health practices. Trained dentists applied the questionnaires through one-to-one interview in the classroom of kindergarten, who helped to read the questionnaire for illiteracy caregivers. The training session were hold by Chinese Stomatological Association and Peking University Hospital of stomatology in Beijing before survey. The oral examination of the children’s dental caries status was performed with the aid of a mirror and a dental probe by three calibrated and accredited dentists with the assist of trained recorders, according to World Health Organization (WHO) criteria [27]. Calibration results were > 0.85 calculated by kappa statistics. The numbers of decayed teeth and missing teeth and filled teeth were recorded based on the criteria recommended by the WHO [27].

### Variables

#### Oral health outcome

Participant children’s oral health was evaluated by dmft index. The dmft index is commonly used for epidemiological studies in dental research [28]. Teeth or filled teeth with caries were recorded as decayed teeth (dt). Missing teeth for whatever reason in children under 9 years old were classified as missing (mt). Filled teeth without secondary caries were classified as filled (ft). The total number of dt, mt and ft. were recorded as dmft [29].

#### Socio-economic status (SES)

SES was measured by caregiver’s education, annual household income and family type [30]. Annual household income was obtained by question that “What is your approximate total household income in the past 12 months?” Caregivers were asked to answer by an exact number. The caregivers were allowed to leave this question unanswered because income is a sensitive issue. As a result, 316 participants were excluded during analysis because of no household income information. Caregivers were required to choose their highest educational attainment from eight options (no formal schooling, primary school, middle school, high school, technical secondary school, junior college, university completed, postgraduate degree or above), which was recorded as the caregiver’s education level. Family type was defined as non-agricultural or agricultural.

#### Knowledge

Caregiver’s oral health knowledge was measured by eight questions [31] as shown in Table 1 and the quality of measurement was shown in Table 2. The correct answer for each question was coded as 1, and incorrect answer or “don’t know” answers were coded as 0. All eight answers were summed to create a single oral health knowledge variable ranging from 0 to 8. Higher scores represent better oral health knowledge.

**Table 1:** Questionnaire about oral health knowledge and oral health attitudes

Number	Question	Answer
Questions about oral health knowledge		
1	Gingival bleeding is normal when toothbrushing	No () Yes () Don’t know ()
2	Germs are one of the reasons for gingivitis	No () Yes () Don’t know ()
3	Toothbrushing is useless to prevent gingivitis	No () Yes () Don’t know ()
4	Dental caries are mainly caused by germs	No () Yes () Don’t know ()
5	Sugar consumption can lead to dental caries	No () Yes () Don’t know ()
6	Fluoride is useless to protect teeth	No () Yes () Don’t know ()
7	Pit and fissure sealant can protect teeth	No () Yes () Don’t know ()
8	Oral disease could influence systemic health	No () Yes () Don’t know ()
Questions about oral health attitudes		
1	My oral health is very important to me	Agree () Disagree () Neither ()
2	Regular dental check-ups are important	Agree () Disagree () Neither ()
3	Teeth condition is decided at birth and is not related to self-care	Agree () Disagree () Neither ()
4	Self-care is important for preventing dental problems	Agree () Disagree () Neither ()
5	It is important to take care of the first molar	Agree () Disagree () Neither ()
6	Mother’s oral health influences children’s oral health	Agree () Disagree () Neither ()

**Table 2:** reliability and validity of knowledge and attitude

Measure	Cronbach Alpha	KMO	*P* of Bartlett Test
knowledge	0.77	0.79	<0.001
attitude	0.73	0.75	<0.001

#### Attitude

Six questions [31] as shown in Table 1 were included as items in the caregiver’s oral health attitude summary score and the quality of measurement was shown in Table 2; the answer for each question was “agree”, “disagree” or “neither”. Responses were coded 1 for a positive attitude and 0 for a negative attitude or neither. The final scores of oral health attitude could range from 0 to 6; higher scores signify a more positive oral health attitude.

#### Dietary behaviours

Consumption of desserts: Frequency of desserts, including cake, bread, biscuits and sweets (several times a day, every day, several times a week, once a week, several times a month, never) [32].

Sugared drink: Frequency of sugared drink, including juice, soda drinks or other soft drinks (several times a day, every day, several times a week, once a week, several times a month, never) [20].

Night eating: “your child usually has sugared drink or desserts before sleeping?” (often, sometimes, never) [33].

#### Tooth brushing behaviours

Tooth brushing: “Does your child brush his teeth daily?” (yes, never) [34].

#### Dental attendance

When was the last time your child visited a dentist?

(My child has never visited a dentist, 1 year ago, within 1 year, within half a year) [27].

#### Gender

Boy or Girl.

### Analysis

Firstly, a descriptive analysis of all variables was done with SPSS for Windows (version 23.0; IBM Corp, Armonk, NY). SEM was performed to test the causal relationships among observed and latent variables using Amos (SPSS plug-in software). In SEM, path analyses with latent variables (PA-LV) were applied, in which Maximum likelihood estimation and bootstrapping were used to fit functions account for the lack of multivariate normality. Two thousand bootstrap samples were re-sampled from the original data set to derive less biased standard errors and 95% percentile confidence interval (CI) and 95% bias-corrected confidence intervals. The model fit was evaluated by various indices used widely in SEM analysis. The model was considered feasible for the analysis only when it achieves the recommended Goodness-of-Fit (GOF) measures. The chi-squared fit statistic (χ^2^/df), root-mean-square error of approximation (RMSEA), GOF index (GFI), normed fit index (NFI) and comparative fit index (CFI) should all be close to or better than the recommended levels when the model is acceptable [35]. In this study, the recommended GOF were used to compare models.

## Results

Descriptive data of variables is shown in Table 3. The final sample was consisted of 2430 children and their caregivers. The mean annual household income is 83 thousand yuan (SD = 87). The mean knowledge score is 4.67(SD = 1.52). The mean dmft is 3.29(SD = 4.13). The relationships between independent factors and children’s dental caries are shown in Fig. 1. GOF measures for the models are shown (Table 4); the χ^2^/df, RMSEA, GFI, NFI, CFI, PGFI, and PNFI of model 1 and model 2 were all close to or better than the recommended fit and model 1 is much better than model 2. Therefore, the hypothetical model 1 was considered suitable for analysing the survey data.

**Table 3:** Descriptive data of variables

Latent variables	Observed variables	n (%) or mean (SD)
SES	Education	
	<Primary school	267 (11%)
	Primary school	713 (29.3%)
	Middle school	791 (32.6%)
	High school or equivalent	405 (16.7%)
	Technical school	161 (6.6%)
	College graduate	88 (3.6%)
	Advance degree	5 (0.2%)
	Household income	83 (87)
	Family type	
	Non-agricultural	755 (31.1%)
	Agricultural	1675 (68.9%)
Knowledge	Knowledge scores	4.67 (1.52)
Attitude	Attitude scores	4.26 (1.16)
Toothbrushing	Toothbrushing	
	Yes	1740 (71.6%)
	Never	690 (28.4%)
dietary behaviour	Dessert	
	Never	114 (4.7%)
	1–3 times/month	207 (8.5%)
	Once/week	194 (8.0%)
	2–6 times/week	617 (25.4%)
	Once/day	807 (33.2%)
	Twice/day	491 (20.2%)
	Sugared drinks	
	Never	569 (23.4%)
	1–3 times/month	247 (10.2%)
	Once/week	170 (7.0%)
	2–6 times/week	493 (20.3%)
	Once/day	702 (28.9%)
	Twice/day	249 (10.2%)
	Night eating	
	Never	353 (14.5%)
	Sometimes	889 (36.6%)
	Often	1188 (48.9%)
Dental attendance	Last time dental attendance	
	Never	2061 (84.8%)
	1 year ago	105 (4.3%)
	within 1 year	108 (4.4%)
	within half year	154 (6.4%)
Dental caries	dmft	3.29 (4.13)

**Table 4:** Goodness of fit measures of SEM model 1 and model 2

Fit index	Index	Recommend levels	Estimate values for hypothesis model	
			Model 1	Model 2
Absolute fit index	c^2^/df	< 5	1.76	1.85
	RMSEA	< 0.08	0.018	0.019
	GFI	> 0.9	0.996	0.994
Incremental fit indices	NFI	> 0.9	0.97	0.96
	CFI	> 0.9	0.987	0.985

*χ*^*2*^*/df* the chi-squared fit statistic, *RMSEA* root-mean-square error of approximation, *GFI* GOF index, *NFI* normed fit index, *CFI* comparative fit index

The hypothetical model 1 presented in Fig. 1 has 5 major components: SES variables, oral health knowledge variables, oral health attitude variables, behavioural variables (dietary behaviours, tooth brushing behaviours and dental attendances) and dental caries variables. Figure 1 provides standardized path coefficient estimates for the proposed model 1. Children who frequently eat desserts and sugared beverages tend to suffer from more dental caries (dmft) (β = 0.11, SE = 0.03, *P* = 0.001), which means that avoiding dessert and sugared beverage intake reduce dental caries for children. Meanwhile, tooth brushing daily did not decrease children’s dental caries (dmft) (β = 0.01, SE = 0.02, *P* = 0.73). At the same time, children from high SES level family are less likely to suffer from dental caries (β = − 0.17, SE = 0.03, *P *<0.001), but dental caries are not directly associated with caregiver’s oral health knowledge (β = − 0.01, SE = 0.03, *P* = 0.68) or oral health attitudes (β = − 0.02, SE = 0.02, *P* = 0.41). Meanwhile, dental caries are positively associated with dental attendance (β = 0.25, SE = 0.02, *P* <0.001), which is an interesting phenomenon in Sichuan provinces that children with more dental attendance are more likely to suffer from dental caries. Oral health behaviors, including dietary behaviors and tooth brushing behaviors and dental attendance were positively associated with SES (β = 0.23, SE = 0.05, *P* <0.001 and β = 0.191, SE = 0.03, *P* <0.001 and β = 0.22, SE = 0.03, *P* <0.001, respectively), means that caregiver with high SES level can master more oral health practices. Additionally, caregivers with positive attitude were not likely to reduce children’s intake sugared food (β = 0.11, SE = 0.04, *P* = 0.002). Daily tooth brushing were positively associated with oral health attitudes (and β = 0.07, SE = 0.02, *P* = 0.003), but not significantly associated with oral health knowledge (β = − 0.01, SE = 0.03, *P* = 0.577). Oral health attitudes were positively linked with both SES (β = 0.18, SE = 0.03, *P* < 0.001) and oral health knowledge (β = 0.31, SE = 0.02, *P* < 0.001). Oral health knowledge was positively associated with SES (β = 0.46, SE = 0.02, *P* < 0.001). Table 5 and Table 6 show the corresponding regression weights and standardized regression weights of the model 1, Table 7 and Table 8 show the corresponding regression weights and standardized regression weights of the model 2.

**Table 5:** regression weight of the model 1

			Estimate	SE	percentile 95%CI	Bia-corrected 95%CI	*P*
Latent variable loadings							
dessert	<---	dietary behaviors	1	1	1	1	
Sugared drink	<---	dietary behaviors	1.932	0.243	1.50/2.56	1.50/2.56	<0.001***
Night eating	<---	dietary behaviors	0.653	0.08	0.51/0.86	0.51/0.87	<0.001***
education	--->	SES	0.274	0.023	0.22/0.33	0.22/0.33	<0.001***
income	--->	SES	1	1	1	1	
Family type	--->	SES	0.05	0.005	0.04/0.06	0.04/0.06	<0.001***
Measured variables							
knowledge	<---	SES	0.213	0.018	0.18/0.26	0.18/0.26	<0.001***
attitude	<---	knowledge	0.236	0.018	0.20/0.27	0.20/0.27	<0.001***
attitude	<---	SES	0.064	0.011	0.04/0.09	0.05/0.09	<0.001***
dietary behaviors	<---	attitude	0.046	0.015	0.02/0.08	0.02/0.08	0.002**
dietary behaviors	<---	knowledge	−0.002	0.012	− 0.03/0.03	− 0.03/0.02	0.885
Toothbrushing	<---	knowledge	−0.004	0.008	−0.02/0.01	− 0.02/0.01	0.577
toothbrushing	<---	attitude	0.026	0.009	0.01/0.04	0.01/0.04	0.003**
toothbrushing	<---	SES	0.026	0.005	0.02/0.04	0.02/0.04	<0.001***
dietary behaviors	<---	SES	0.034	0.008	0.02/0.05	0.02/0.05	<0.001***
Dental attendance	<---	SES	0.055	0.009	0.04/0.07	0.04/0.08	<0.001***
Dental attendance	<---	knowledge	−0.007	0.014	−0.03/0.02	−0.04/0.02	0.635
Dental attendance	<---	attitude	0.014	0.016	−0.01/0.04	−0.01/0.04	0.389
dmft	<---	SES	−0.208	0.046	−0.31/− 0.13	−0.31/− 0.13	<0.001***
dmft	<---	knowledge	−0.028	0.068	−0.17/0.11	−0.17/0.11	0.68
dmft	<---	attitude	−0.065	0.08	−0.22/0.10	−0.23/0.08	0.41
dmft	<---	dietary behaviors	0.945	0.295	0.36/1.59	0.38/1.61	0.001**
dmft	<---	toothbrushing	0.066	0.188	−0.31/0.45	−0.31/0.46	0.73
dmft	<---	dental attendance	1.253	0.102	1.02/1.50	1.01/1.49	<0.001***

*<0.05, **<0.01, ***<0.001

**Table 6:** standardized regression weight of the model 1

			Estimate	SE	percentile 95%CI	Bia-corrected 95%CI	*P*
Latent variable loadings							
dessert	<---	dietary behaviors	0.35	0.03	0.29/0.42	0.29/0.42	<0.001***
sugared drink	<---	dietary behaviors	0.53	0.04	0.46/0.61	0.45/0.61	<0.001***
night eating	<---	dietary behaviors	0.44	0.03	0.37/0.50	0.37/0.50	<0.001***
education	--->	SES	0.73	0.03	0.68/0.79	0.68/0.79	<0.001***
income	--->	SES	0.38	0.03	0.33/0.44	0.32/0.44	<0.001***
Family type	--->	SES	0.35	0.03	0.30/0.40	0.31/0.40	<0.001***
Measured variables							
knowledge	<---	SES	0.46	0.02	0.41/0.50	0.41/0.50	<0.001***
attitude	<---	knowledge	0.31	0.02	0.26/0.36	0.26/0.36	<0.001***
attitude	<---	SES	0.18	0.03	0.13/0.23	0.13/0.23	<0.001***
dietary behaviors	<---	attitude	0.11	0.04	0.04/0.18	0.04/0.18	0.002**
dietary behaviors	<---	knowledge	−0.01	0.04	− 0.08/0.07	− 0.08/0.08	0.885
toothbrushing	<---	knowledge	− 0.01	0.03	−0.06/0.03	− 0.06/0.03	0.577
toothbrushing	<---	attitude	0.07	0.02	0.03/0.11	0.02/0.11	0.003**
toothbrushing	<---	SES	0.19	0.03	0.13/0.25	0.13/0.25	<0.001***
dietary behaviors	<---	SES	0.23	0.05	0.14/0.32	0.14/0.32	<0.001***
Dental attendance	<---	SES	0.22	0.03	0.15/0.28	0.15/0.29	<0.001***
Dental attendance	<---	knowledge	−0.01	0.03	−0.06/0.04	− 0.06/0.04	0.635
Dental attendance	<---	attitude	0.02	0.02	−0.02/0.06	−0.02/0.06	0.389
dmft	<---	SES	−0.17	0.03	−0.23/− 0.10	−0.23/− 0.10	<0.001***
dmft	<---	knowledge	−0.01	0.03	−0.06/0.04	− 0.06/0.04	0.68
dmft	<---	attitude	−0.02	0.02	−0.06/0.03	−0.06/0.02	0.41
dmft	<---	Dietary behaviors	0.11	0.03	0.04/0.17	0.05/0.18	0.001**
dmft	<---	toothbrushing	0.01	0.02	−0.03/0.05	−0.03/0.05	0.73
dmft	<---	dental attendance	0.25	0.02	0.21/0.30	0.21/0.30	***

*<0.05, **<0.01, ***<0.001

**Table 7:** Regression weight of the model 2

			Estimate	SE	percentile 95%CI	Bia-corrected 95%CI	*P*
Latent variable loadings							
dessert	<---	dietary behaviors	1		1	1	
Sugared drink	<---	dietary behaviors	1.94	0.25	1.51/2.09	1.50/2.58	<0.001***
Night eating	<---	dietary behaviors	0.65	0.08	0.50/0.86	0.50/0.86	<0.001***
education	<---	SES	1		1	1	
income	<---	SES	3.62	0.3	2.99/4.41	3.00/4.43	<0.001***
Family type	<---	SES	0.18	0.02	0.15/0.22	0.15/0.24	<0.001***
Measured variables							
dental attendance	<---	SES	0.08	0.01	0.06/0.11	0.06/0.11	<0.001***
knowledge	<---	SES	0.77	0.06	0.65/0.89	0.65/0.90	<0.001***
knowledge	<---	dental attendance	0.06	0.09	−0.12/0.22	− 0.12/0.21	0.54
attitude	<---	knowledge	0.24	0.02	0.20/0.27	0.20/0.27	<0.001***
attitude	<---	SES	0.23	0.04	0.15/0.30	0.16/0.31	<0.001***
attitude	<---	dental attendance	0.06	0.06	−0.05/0.16	− 0.05/0.16	0.36
dietary behaviors	<---	attitude	0.05	0.02	0.02/0.08	0.02/0.08	0.003**
toothbrushing	<---	attitude	0.03	0.01	0.01/0.04	0.01/0.04	0.002**
toothbrushing	<---	SES	0.09	0.02	0.06/0.12	0.06/0.12	<0.001***
dietary behaviors	<---	SES	0.13	0.03	0.07/0.19	0.07/0.19	<0.001***
dietary behaviors	<---	dental attendance	−0.03	0.04	−0.11/0.07	− 0.11/0.07	0.58
toothbrushing	<---	dental attendance	0.04	0.03	−0.01/0.09	−0.01/0.09	0.13
dietary behaviors	<---	knowledge	0	0.01	−0.03/0.02	−0.03/0.02	0.88
toothbrushing	<---	knowledge	0	0	−0.02/0.01	−0.02/0.01	0.64
dmft	<---	attitude	− 0.07	0.08	−0.22/0.09	− 0.23/0.08	0.4
dmft	<---	dietary behaviors	0.93	0.29	0.38/1.57	0.40/1.59	0.001**
dmft	<---	toothbrushing	0.04	0.19	−0.34/0.43	− 0.33/0.43	0.83
dmft	<---	dental attendance	3.22	0.24	2.66/3.78	2.63/3.76	<0.001***
dmft	<---	SES	−0.76	0.17	−1.13/− 0.45	−1.14/− 0.46	<0.001***
dmft	<---	knowledge	− 0.04	0.07	− 0.18/0.09	− 0.18/0.09	0.51

*<0.05, **<0.01, ***<0.001

**Table 8:** standardized regression weight of the model 2

			Estimate	SE	percentile 95%CI	Bia-corrected 95%CI	*P*
Latent variable loadings							
dessert	<---	dietary behaviors	0.351	0.034	0.28/0.42	0.28/0.42	<0.001***
Sugared drink	<---	dietary behaviors	0.532	0.041	0.46/0.62	0.46/0.61	<0.001***
Night eating	<---	dietary behaviors	0.433	0.034	0.37/0.50	0.37/0.50	<0.001***
education	<---	SES	0.734	0.03	0.68/0.80	0.68/0.80	<0.001***
income	<---	SES	0.353	0.03	0.30/0.40	0.30/0.40	<0.001***
Family type	<---	SES	0.375	0.03	0.33/0.44	0.32/0.43	<0.001***
Measured variables							
dental attendance	<---	SES	0.212	0.027	0.16/0.27	0.16/0.27	<0.001***
knowledge	<---	SES	0.453	0.025	0.40/0.50	0.40/0.50	<0.001***
knowledge	<---	dental attendance	0.013	0.019	−0.03/0.05	−0.03/0.05	0.54
attitude	<---	knowledge	0.31	0.023	0.27/0.36	0.27/0.36	<0.001***
attitude	<---	SES	0.177	0.028	0.12/0.23	0.12/0.23	<0.001***
attitude	<---	dental attendance	0.018	0.017	−0.02/0.05	−0.02/0.05	0.36
dietary behaviors	<---	attitude	0.111	0.035	0.04/0.18	0.04/0.18	0.003**
toothbrushing	<---	attitude	0.068	0.023	0.03/0.11	0.03/0.11	0.002**
toothbrushing	<---	SES	0.175	0.031	0.12/0.24	0.12/0.24	<0.001***
dietary behaviors	<---	SES	0.24	0.051	0.14/0.36	0.14/0.34	<0.001***
dietary behaviors	<---	dental attendance	−0.018	0.034	−0.08/0.05	−0.08/0.05	0.58
toothbrushing	<---	dental attendance	0.031	0.02	−0.01/0.07	−0.01/0.07	0.13
dietary behaviors	<---	knowledge	−0.006	0.04	−0.08/0.08	−0.08/0.08	0.88
toothbrushing	<---	knowledge	−0.012	0.025	−0.06/0.04	−0.06/0.04	0.64
dmft	<---	attitude	−0.019	0.022	−0.06/0.03	−0.07/0.02	0.4
dmft	<---	dietary behaviors	0.108	0.033	0.04/0.17	0.05/0.17	0.001**
dmft	<---	toothbrushing	0.004	0.021	−0.04/0.05	−0.04/0.05	0.83
dmft	<---	dental attendance	0.28	0.024	0.23/0.33	0.23/0.33	<0.001***
dmft	<---	SES	−0.166	0.034	−0.23/− 0.10	−0.23/− 0.10	<0.001***
dmft	<---	knowledge	−0.016	0.025	−0.07/0.03	−0.07/0.03	0.51

*<0.05, **<0.01, ***<0.001

The indirect and total effects of 5 major variables of the model were examined, and these results are presented in Table 9. The direct effect of SES on dmft is negative significantly (β = − 0.17, SE = 0.03, BC 95% CI = -0.23/− 0.10), While the indirect effect of SES on dmft is in an opposite direction(β = 0.08,SE = 0.02, BC 95% CI = 0.04/0.12),Which means that the indirect link from SES-knowledge-attitude-behaviours-dmft is broken at some point.

**Table 9:** Total effect, direct effect and indirect of the variables of model 1

				estimate	SE	Bia-corrected 95%CI	percentile 95%CI
attitude	<---	SES	Total	0.32	0.02	0.27/0.37	0.27/0.37
			indirect	0.14	0.01	0.12/0.17	0.12/0.17
			direct	0.18	0.03	0.13/0.23	0.13/0.23
toothbrushing	<---	SES	Total	0.20	0.03	0.16/0.25	0.16/0.25
			indirect	0.02	0.01	− 0.01/0.04	−0.01/0.04
			direct	0.19	0.03	0.13/0.25	0.13/0.25
dietary behaviors	<---	SES	Total	0.27	0.04	0.19/0.35	0.19/0.34
			indirect	0.03	0.02	0.00/0.07	0.00/0.07
			direct	0.23	0.05	0.14/0.32	0.14/0.32
dental attendance	<---	SES	Total	0.22	0.03	0.16/0.27	0.16/0.27
			indirect	0	0.01	−0.02/0.02	− 0.02/0.02
			direct	0.22	0.03	0.15/0.29	0.15/0.28
dmft	<---	SES	Total	−0.09	0.03	− 0.14/− 0.04	−0.14/− 0.04
			indirect	0.08	0.02	0.04/0.12	0.04/0.12
			direct	−0.17	0.03	− 0.23/− 0.10	−0.21/− 0.07
toothbrushing	<---	knowledge	Total	0.02	0.03	−0.04/0.06	−0.14/− 0.04
dietary behaviors	<---	knowledge	Total	0.03	0.04	−0.04/0.11	−0.05/0.11
dmft	<---	knowledge	Total	−0.01	0.03	−0.07/0.03	−0.07/0.04
dmft	<---	attitude	Total	−0.01	0.02	−0.05/0.04	−0.04/0.04

The total effect of SES on oral health attitude is positively significant (β = 0.32, SE = 0.02, BC 95% CI = 0.27/0.37), in which indirect effect of SES on oral health attitude through is a positive link (β = 0.14, SE = 0.01, BC 95% CI = 0.12/0.17), which means the caregiver with better household SES tend to obtain positive oral health attitude. The total association of SES to dietary behaviors is opposite of the hypothesized direction (β = 0.27, SE = 0.04, BC 95% CI = 0.19/0.35) as well as the indirect effect (β = 0.03, SE = 0.02, BC 95% CI = 0.00/0.07) and direct effect (β = 0.23, SE = 0.05, BC 95% CI = 0.14/0.32). Which means that caregivers with better SES were not more likely to avoid sugared beverage intake. SES effect on tooth brushing behaviours positive directly (β = 0.19, SE = 0.03, BC 95% CI = 0.13/0.25) but not significant for indirect (β = 0.02, SE = 0.01, BC 95% CI = -0.01/0.04). So far we narrow the broken point into oral health knowledge-oral health attitude-behaviours. The total effect of knowledge on both dietary behaviours and toothbrushing behaviours are not significant (β = 0.03, SE = 0.04, BC 95% CI = -0.04/0.11 and β = 0.02, SE = 0.03, BC 95% CI = -0.04/0.06, respectively). Given that direct effect of attitude on dietary behaviours is opposite to the hypothesis direction mentioned above. The link oral health knowledge and attitude with oral health behaviours is separation, which lead to the indirect effect of SES on dmft is not significant.

## Discussion

This study provides comprehensive information about factors associated with dental caries in 3–5 years old children in Sichuan Province. Dietary behaviours were also directly associated with dental caries. The high caries experience was more commonly seen in children who often pick sugared beverage and dessert, and who have night eating behaviour; previous study was also in accordance with that [36]. Therefore, warning labels pertaining sugar intake is necessary. And, it is also important for clinicians to analysis children’ dietary behaviours and give advice about dietary in treatment plans.

Here SES is directly associated with children’s dmft, children from higher income and education families had a significantly lower chance to suffer dental caries. These findings were consistent with previous studies [37]. However, SES is positively associated with dietary behaviours, the potential reason to interpret this phenomenon is that family with high income and education do not realize the risk of sugared beverage and dessert intake. Parents need to have better oral health education about feeding.

Both SES and attitude are positively linked with toothbrushing, but toothbrushing isn’t significant with the prevalence of dental caries. However toothbrushing is the recommended oral hygiene methods to prevent dental caries have been widely proven. This finding indicates that children in Sichuan provinces don’t brush their teeth with rightly method. In our study, only 57.7% of children among those who brush their teeth frequently have ever brushed teeth under a caregiver’s supervision, which cannot guarantee the effectiveness of toothbrushing. Proper technique of brushing teeth such as Bass Method should be widely publicize [38]. Parents should remind their child to brush daily and help their child to brush their teeth before the recommended age [39].

Dental caries were significantly associated with oral health behaviours and SES directly. However, the indirect link through traditional oral health education link SES-knowledge-attitude-behaviour pathway did not influence dmft. Figure out the vulnerable spot can help the planning and evaluation of the oral health promotion program for children 3–5 years old in Sichuan province. In this study, SES is positively associated with oral health knowledge and attitude. Generally speaking, people with high SES will have much more opportunity to access information about health, which is consistent with previous study [11]. We noticed that SES influence attitude through knowledge, which means our oral health knowledge education is effective. A study in Guangzhou in 2014 [11] found that caregivers with better SES were equipped with better oral health knowledge and attitudes. The link from dietary behaviours to dmft is also significant. Therefore, vulnerable spot in the whole SES-knowledge-attitude-behaviours-dmft should be located in the knowledge-attitude-behaviours part. The indirect effect of SES on dietary behaviours through oral health knowledge and attitude is opposite with hypothesis association. The indirect effect of SES on toothbrushing through oral health knowledge and attitude is not significant. The total effect of knowledge on oral health behaviours (dietary behaviours and toothbrushing behaviours) isn’t significant. So we found that the association between oral health knowledge, attitude and oral health behaviours is separated. In the present study, oral health behaviours were not directly or indirectly associated with oral health knowledge. This is consistent with findings from a Singapore study that questioned the theoretical assumption of the knowledge-attitude-practice (KAP) links and the notion that health improvements are achievable through professional health education [15]. This finding recommends that specialists should rethink about health education contents. Health educators should educate and motivate caregivers with specific advice and information triggering corresponding practices instead of giving general oral health knowledge. A layperson does not necessarily have knowledge about how germs causing dental caries and how fluoride prevents tooth decay.

Dental clinic attendance was positively related to dental caries, which indicated abnormal phenomenon in Sichuan provinces. Children were more likely to use dental care services for tooth pain instead of regular checkups, which have been reported in previous studies in another region [40]. Therefore, children using dental care services were more likely to experience dental caries. It is necessary to emphasize the important of regular check-up and oral preventive care.

The greatest strength of our study lies in the integration of various factors with untreated dental caries by SEM, in which superior and multiple regression modelling can show direct relationships between one oral health outcome and various risk factors. SEM was first used in the field of social sciences and has become popular in dental sciences [40–42]. SEM is a multivariate statistical method able to evaluate a network of relationships between observed and latent variables, and measure the overall model fit [43]. Model fit to the data was assessed with various indices used widely in SEM analysis. To our knowledge, this was the first SEM model applied to data from Sichuan Province. The second strength is that our sample size is sufficiently large enough to represent the entire population of Sichuan Province.

This study has limitations. First, dental caries is caused by multiple factors, which contain physical, biological, environmental, behaviour and lifestyle-related factors such as high numbers of cariogenic bacteria, inadequate salivary flow, insufficient fluoride exposure, poor oral hygiene, inappropriate methods of feeding infants and poverty [2]. We only included some of these risk factors in our study. Second, this study was a cross-sectional study; however, dental caries is a chronic and progressive disease better suited to longitudinal research.

## Conclusion

SES and dietary behaviour variables play a crucial role in explaining dental caries outcomes, children from high level SES family were more likely to suffer from dental caries.

Generally, good oral health knowledge and positive oral health attitude can improve oral health, however, oral health knowledge and attitude failed to affect dietary behaviour in this model, meanwhile we find that unhealthy dietary behaviour can lead to an increase in caries. These results demonstrate that the knowledge of healthy feeding is not propagated enough in Sichuan province.

SES affects oral health knowledge and attitude, but oral health knowledge and attitude failed to affect dietary behaviour in our research. This founding warns that it is easier than done. Future oral health education should focus on finding a more effective way for the public to turn knowledge into action. A policy for dental caries prevention should focus on effective oral health education and triggering corresponding protective oral health behaviours.

## Abbreviations

CFI: Comparative fit index
GFI: GOF index
GOF: Goodness-of-Fit
NFI: Normed fit index
PA-LV: Path analyses with latent variables
PPS: Probabilities proportional to size
RMSEA: Root-mean-square error of approximation
SEM: Structural equation modelling
SES: Socio-economic status
χ^2^/df: Chi-squared fit statistic
